# Enhancing Multi-Center Generalization of Machine Learning-Based Depression Diagnosis From Resting-State fMRI

**DOI:** 10.3389/fpsyt.2020.00400

**Published:** 2020-05-28

**Authors:** Takashi Nakano, Masahiro Takamura, Naho Ichikawa, Go Okada, Yasumasa Okamoto, Makiko Yamada, Tetsuya Suhara, Shigeto Yamawaki, Junichiro Yoshimoto

**Affiliations:** ^1^Division of Information Science, Graduate School of Science and Technology, Nara Institute of Science and Technology, Ikoma, Japan; ^2^Department of Psychiatry and Neurosciences, Hiroshima University, Hiroshima, Japan; ^3^Institute of Quantum Life Science, National Institutes for Quantum and Radiological Science and Technology, Chiba, Japan; ^4^Department of Functional Brain Imaging, National Institute of Radiological Sciences, National Institutes for Quantum and Radiological Science and Technology, Chiba, Japan

**Keywords:** depression, functional connectivity, machine learning, harmonization, multi-center fMRI, resting state fMRI

## Abstract

Resting-state fMRI has the potential to help doctors detect abnormal behavior in brain activity and to diagnose patients with depression. However, resting-state fMRI has a bias depending on the scanner site, which makes it difficult to diagnose depression at a new site. In this paper, we propose methods to improve the performance of the diagnosis of major depressive disorder (MDD) at an independent site by reducing the site bias effects using regression. For this, we used a subgroup of healthy subjects of the independent site to regress out site bias. We further improved the classification performance of patients with depression by focusing on melancholic depressive disorder. Our proposed methods would be useful to apply depression classifiers to subjects at completely new sites.

## Introduction

Depressive disorder is a mental disorder characterized by long-lasting low mood. The diagnosis of depressive disorder has traditionally been made through the interaction between patients and doctors. It is important to develop more objective ways to diagnose depressive disorder in order to increase the reliability and accuracy of the diagnosis.

The combination of machine learning and functional magnetic resonance imaging (fMRI) has been used to diagnose or to find the physiological characteristics of psychiatric disorders (1–11). Recently, functional connectivity (the correlation coefficients of the brain activity between brain regions) easily calculated from resting-state fMRI data are being used for the diagnosis of psychiatric disorders, such as autism, obsessive-compulsive disorder, and depression (1–7). For resting-state fMRI, spontaneous brain activity was measured from subjects lying in an fMRI scanner without any stimulation. Because of the advantages of resting-state fMRI, functional connectivity has the potential to become a clinical tool widely used in hospitals.

However, functional connectivity is affected by site bias (2, 12). To overcome site bias, some studies use independent component analysis or sparse canonical component analysis to extract site independent components (7, 13). There are also some harmonization methods using regression out procedure, such as combat, traveling subject methods, and generalized linear model (GLM) methods (1, 2, 14–18). Another approach that can be effective is using data from as many sites as possible for the training of classification algorithms. These approaches reduced the bias of the site where data is already available. Even though data from many sites were used for machine learning, it is difficult to apply this to a completely new site data.

In addition to site bias, the heterogeneity of major depressive disorder would be a problem when the classifier is applied to new data. The abnormality of functional connectivity of depression might be different depending on the subtype of the depression. It would be possible to improve classification performance by focusing on a typical severe depression, called melancholic depressive disorder. In this paper, we propose the methods to improve the performance of diagnosis of major depressive disorder (MDD) at an independent site by reducing the site bias effects. In addition, we investigated the performance depending on the classification algorithms and the heterogeneity of the major depressive disorder.

## Materials and Methods

### Subjects

One hundred sixty-three patients with MDD (age 20-75, average 44.1 ± 12.2) were recruited by five sites (the Psychiatry Department of Hiroshima University and collaborating medical institutions, **Table 1**). They were screened using the Mini International Neuropsychiatric Interview (M.I.N.I), (19, 20), which enables medical doctors to identify psychiatric disorders according to DSM-IV criteria (21). Patients had an initial MRI scan before or within two weeks after starting medication of selective serotonin reuptake inhibitors (SSRIs).

**Table 1:** Demographic data of study participants.

Site 1 (HUH: Hiroshima University Hospital)			
	HC	MDD	p value
No. of participants (Male/Female)	59 (26/33)	59 (32/27)	
No. of melancholia (Male/Female)	NA	49 (29/20)	
Age (years)	33.7 ± 12.5	42.8 ± 12.0	<0.001
Severity of depression (BDI-II)	6.9 ± 5.9	30.1 ± 8.5	<0.001
IQ (JART)	113.7 ± 8.3	108.7 ± 9.7	0.004
**Site 2 (HRC: Hiroshima Rehabilitation Center)**			
	HC	MDD	p value
No. of participants (Male/Female)	12 (3/9)	12 (6/6)	
No. of melancholia (Male/Female)	NA	6 (0/6)	
Age (years)	42.4 ± 9.4	41.8 ± 10.4	0.667
Severity of depression (BDI-II)	11.0 ± 12.6	35.3 ± 10.0	<0.001
IQ (JART)	111.5 ± 5.8	120.3 ± 5.1	<0.001
**Site 3 (HKH: Hiroshima Kajikawa Hospital)**			
	HC	MDD	p value
No. of participants (Male/Female)	22 (5/17)	22 (12/10)	
No. of melancholia (Male/Female)	NA	14 (8/6)	
Age (years)	46.4 ± 9.4	44.0 ± 11.7	0.497
Severity of depression (BDI-II)	5.5 ± 5.3	29.8 ± 7.1	<0.001
IQ (JART)	115.3 ± 5.7	117.8 ± 5.5	0.178
**Site 4 (COI: Center of Innovation in Hiroshima University)**			
	HC	MDD	p value
No. of participants (Male/Female)	55 (20/35)	55 (24/31)	
No. of melancholia (Male/Female)	NA	25 (11/14)	
Age (years)	53.7 ± 12.2	47.2 ± 13.0	0.011
Severity of depression (BDI-II)	8.5 ± 6.6	25.7 ± 9.6	<0.001
IQ (JART)	107.4 ± 11.5	112.2 ± 10.4	0.020
**Site 5 (QST: National Inst. for Quantum and Radiological Sci.and Tech.)**			
	HC	MDD	p value
No. of participants (Male/Female)	47 (41/6)	15 (9/6)	
No. of melancholia (Male/Female)	NA	11 (6/5)	
Age (years)	24.4 ± 5.8	39.7 ± 10.3	<0.001
Severity of depression (BDI-II)	4.6 ± 4.2	28.8 ± 10.2	<0.001
IQ (JART)	–	–	–

HC, healthy control group; MDD, major depressive disorder patient group. BDI-II, Beck Depression Inventory-II; JART, Japanese Adult Reading Test.

Values are presented as mean ± s.d.

As a control group, 195 healthy subjects (age 20-75, average 39.1 ± 15.5) with no history of mental or neurological disease were recruited from the local community. All control subjects underwent the same self-assessment and examination administered to the MDD group. Thereafter, for the melancholic MDD classifier, the dataset was limited to have the subtype of melancholia (based on M.I.N.I.). The number of patients and healthy controls were set to be equal for each site, except for the subjects from the National Institutes for Quantum and Radiological Science and Technology, in order to develop a classifier unbiased toward either group (see **Table 1**). The subjects from the National Institutes for Quantum and Radiological Science and Technology were used only for the test data (replication cohort).

The study protocol in this study was approved by the Ethics Committee of Hiroshima University and the Radiation Drug Safety Committee and by the institutional review board of the National Institutes for Quantum and Radiological Science and Technology, in accordance with the ethical standards laid down in the 1964 Declaration of Helsinki and its later amendments.

The detailed properties of the subjects are shown in **Table 1**.

### Acquisition and Preprocessing of Functional MRI Data

fMRI scanners were used to generate magnetic resonance images. Functional data were collected using gradient echo planar imaging (EPI) sequences. High-resolution T1-weighted magnetization-prepared rapid gradient echo images were also acquired before scanning the functional data. In the scan room with dimmed lights, participants were instructed not to think of anything in particular, not to sleep, and to keep looking at a cross mark at the center of the monitor screen. The first four to seven images were discarded to allow magnetization to reach equilibrium. All participants underwent an approximately 5 to 10 min resting-state scan. The scanners and imaging parameters are different depending on the site (see **Tables 1** and **2**).

**Table 2:** Imaging protocols for resting-state fMRI.

	Discovery cohort				Replication cohort
Site	Site 1 (HUH)	Site 2 (HRC)	Site 3 (HKH)	Site 4 (COI)	Site 5 (QST)
MRI scanner	GE SignaHD x t	GE SignaHD x t	Siemens Spectra	Siemens Verio	Siemens Verio
Magnetic field strength (T)	3.0	3.0	3.0	3.0	3.0
Number of channels per coil	8	8	12	12	32
Field of view (mm)	256 x 256	256 x 256	192 x 192	212 x 212	240 x 240
Matri x	64 x 64	64 x 64	64 x 64	64 x 64	64 x 64
Number of slices	32	32	38	40	33
Number of volumes	143	143	107	240	204
In-plane resolution (mm)	4.0 x 4.0	4.0 x 4.0	3.0 x 3.0	3.3125 × 3.3125	3.75 x 3.75
Slice tickness (mm)	4.0	4.0	3.0	3.2	3.8
Slice gap (mm)	0.0	0.0	0.0	0.8	0.5
TR (ms)	2.0	2.0	2.7	2.5	2.0
TE (ms)	27.0	27.0	31.0	30.0	25.0
Total scan time (mm:ss)	5:00	5:00	5:00	10:00	6:52
Flip angle (deg)	90	90	90	80	90
Slice acquision order	Ascending (Interleaved)	Ascending (Interleaved)	Ascending (Interleaved)	Ascending	Ascending (Interleaved)

All the resting-state fMRI data were preprocessed using the identical procedures described in (7). T1-weighted structural image and resting-state functional images were preprocessed using SPM8 (Wellcome Trust Centre for Neuroimaging, University College London, UK) on Matlab R2014a (Mathworks inc., USA). The functional images were preprocessed with slice-timing correction and realignment to the mean image. Thereafter, using the normalization parameters obtained through the segmentation of the structural image aligned with the mean functional image, the fMRI data was normalized and resampled in 2 x 2 x 2 mm^3^ voxels. Finally, the functional images were smoothed with an isotropic 6mm full-width half-maximum Gaussian kernel.

Then potential confounding effects (i.e. the temporal fluctuations of the white matter, the cerebrospinal fluid, and the entire brain as well as six head motion parameters) were linearly regressed out from the fMRI time series (22, 23). Here, the fluctuation in each tissue class was determined from the average time course of the voxels within a mask created by the segmentation procedure of the T1 image. The mask for the white matter was eroded by one voxel to consider a partial volume effect. After these preprocessing steps, the scrubbing procedure (24) was performed to exclude any volume (i.e., functional image) with excessive head motions, based on the frame-to-frame relative changes in time series data.

### Calculation of Functional Connectivity

For each individual, the time course of fMRI data was extracted for each of the 137 regions of interest (ROIs), anatomically defined in the Brainvisa Sulci Atlas (BSA; http://brainvisa. Info) (6, 24) covering the entire cerebral cortex without a cerebellum. After applying a band-pass filter (0.008–0.1 Hz), the following nine parameters were linearly regressed out: the six head motion parameters from realignment, the temporal fluctuation of the white matter, that of the cerebrospinal fluid, and that of the entire brain. Pair-wise Pearson correlations between 137 ROIs were calculated to obtain a matrix of 9,316 functional connectivities for each participant.

### Data Standardization, Removal of Nuisance Variables, and Feature Selections

**Figure 1:**
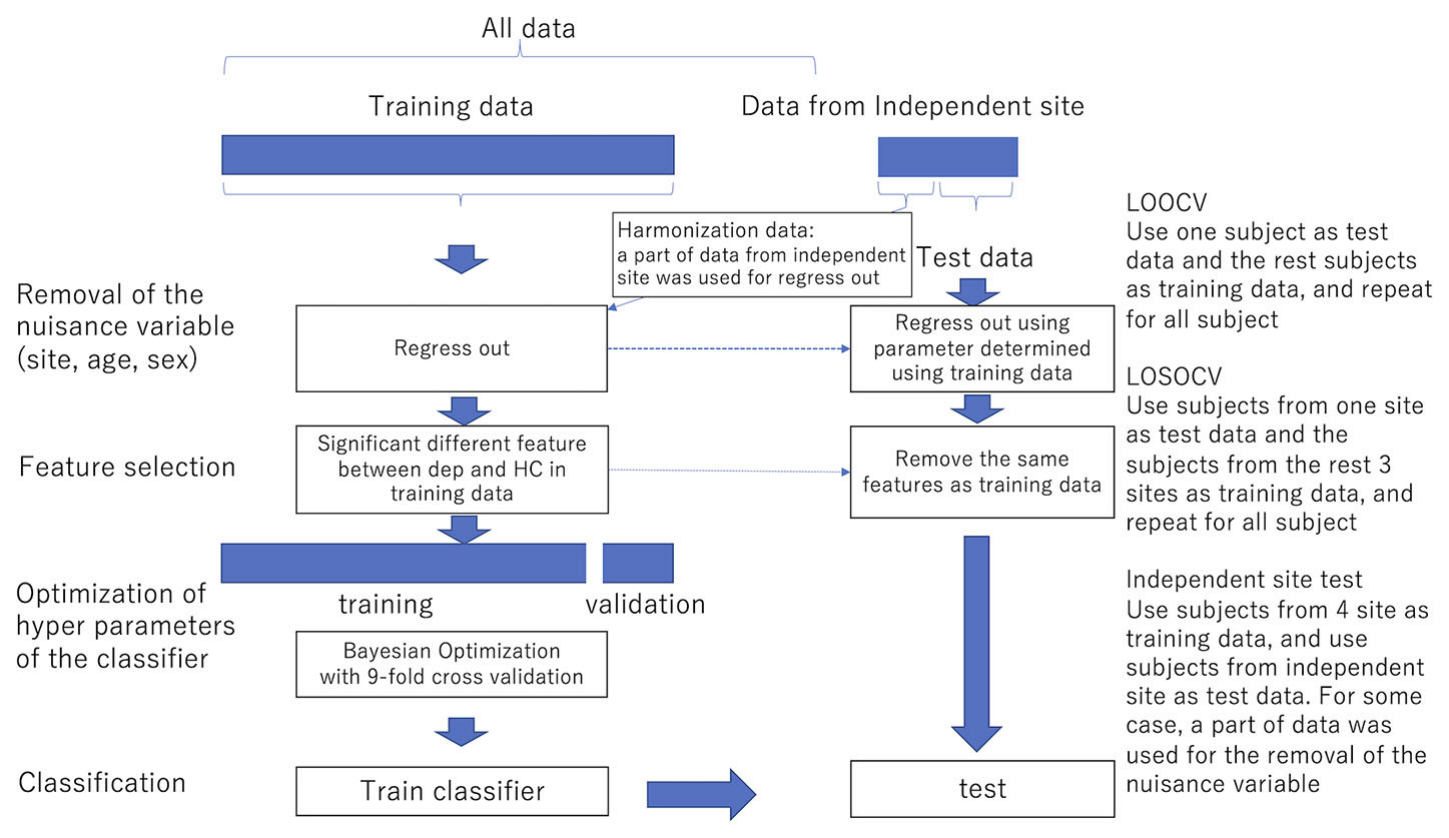
The procedure of the removal of site bias and feature selection. LOOCV Use one subject as test data and the remaining subjects as training data, and repeat for all subjects LOSOCV Use subjects from one site as test data and subjects from the remaining three sites as training data, and repeat for all subjects Independent site test Use subjects from four sites as training data and use subjects from the independent site as test data. In some cases, a part of data was used for the removal of nuisance variables.

For functional connectivity data, we applied the Fisher z-transformation to each correlation coefficient. We then linearly regressed them on training subjects' ages, sex, and dummy variables for each site. The resulting residuals of the regression are used for the subsequent procedures as the features controlling for age, sex, and site effects. We also used the regression coefficients to remove nuisance variables such as age, sex, and site from test subjects. Features like functional connectivities were high-dimensional. We then used a rank-sum test for dimensional reduction; we used the threshold of 0.05 unless noted. The procedures are summarized in **Figure 1**.

### Classification Algorithms

We then applied machine learning classification using the above features. We used ensemble learning (Random Forest and AdaBoost), support vector machine (SVM), or sparse logistic regression (SLR) (25) to classify depressed patients and healthy controls, or melancholic patients and healthy controls. Ensemble learning is a method to make a classifier by combining weak classifiers. While Random Forest makes the decision based on voting by parallel weak classifiers, AdaBoost combines weak classifiers sequentially.

We implemented both ensemble learning methods using MATLAB fitcensemble function and each decision tree for the weak classifier was constructed using the standard CART algorithm (26).

The hyperparameters adjusting tree depth (minimum leaf size and the maximum number of splits), and the other hyperparameters such as the number of ensemble learning cycles and learning rates were optimized using Bayesian optimization described in the following section. Ensemble learning has advantages in dealing with small sample size, and high-dimensionality (27). SVM is a widely used classifier and performs classification by finding the hyperplane that differentiates the two classes. Types of the kernel and penalty parameter were determined by the optimization in the following section. SLR is a Bayesian extension of logistic regression in which a sparseness prior is imposed on the logistic regression. SLR has the ability to select features objectively that are related to classifying depressive disorders (2, 7, 25).

### Evaluation and Estimation of the Hyperparameters of Classifiers

The performance of the classification was evaluated using Leave-One-Subject-Out cross-validation, Leave-One-Site-Out cross-validation within the discovery cohort or an independent dataset from a replication cohort.

For Leave-One-Subject-Out cross-validation, one subject was used for the test and the rest of the subjects were used for the training and this process was repeated until all subjects were used as test data. The hyperparameters of the AdaBoost (learn rate, minimum observations per leaf, maximal number of decision splits, and number of ensemble learning cycles), Random Forest (minimum observations per leaf, maximal number of decision splits, and number of ensemble learning cycles), and SVM (box constraint (a regularization factor (often denoted as parameter C), controlling maximum penalty imposed on margin-violating observations), kernel scale (a parameter to scale the input features), and kernel functions (gaussian, linear or polynomial) were optimized with Bayesian optimization using the Gaussian process with Expected Improvement acquisition function (28). Random Forest, Adaboost, and SVM were implemented by using MATLAB's fitcensemble and fitcsvm functions. Bayesian optimization was implemented by OptimizeHyperparameters option in these functions with default parameters.

For the optimization, the whole data was divided into 9-folds, keeping an equal amount of diagnosis, gender, and site combinations per fold. The optimization was performed using the 8-folds with 8-folds cross-validation (the inner 7-folds are used for the training and the remaining inner one-fold was used for the validation). The test data was selected in the outer 1-fold. The rest from outer 1-fold and the outer 8-folds were used for the training of the classifier (7).

For Leave-One-Site-Out cross-validation, subjects from three out of the four sites were used as the training data and subjects from the remaining sites were used as test data. In the case that an independent dataset was used as a test data, all subjects from the four sites were used for training. For both cases, the optimization of the hyperparameters was done using 8-folds cross-validation within the training data.

## Results

**Figure 2:**
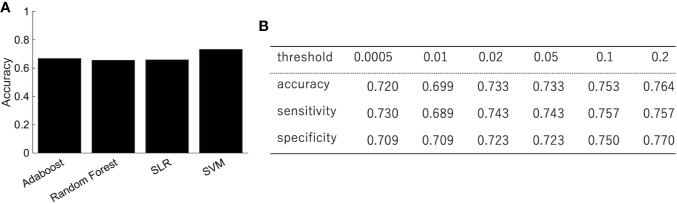
The performance of the classification of depressed patients. **(A)** The SVM shows an accuracy of 73.3%, sensitivity of 74.3%, and specificity of 72.3%. **(B)** The classification performance with different threshold of feature selection.

We trained the classifier of the major depressive disorder using functional connectivity as the features. Because of the high dimensionality of functional connectivity, we selected the features using a rank-sum test with only the training data. **Figure 2A** shows the classification performance of AdaBoost, Random Forest, SLR, and SVM with 0.05 threshold of the rank-sum test. While SVM shows the best classification accuracy, SLR did not show good accuracy. Then we focused on the SVM (the best classifier in **Figure 2A**) and investigated the effect of the threshold for the feature selection in terms of three indexes: accuracy, sensitivity (the number of subjects classified as patients divided by the number of patients), and specificity (the number of subjects classified as healthy controls divided by the number of healthy controls). The best performance was achieved (accuracy = 73.3%; sensitivity = 74.3%; and specificity =72.3%) when the threshold for the feature selection was 0.05 (**Figure 2B**). More remarkably, the performance was fairly stable even if we changed the threshold in the range between 0.0005 and 0.2, and the tradeoff between sensitivity and specificity was always solved well (**Figure 2B**).

**Figure 3:**
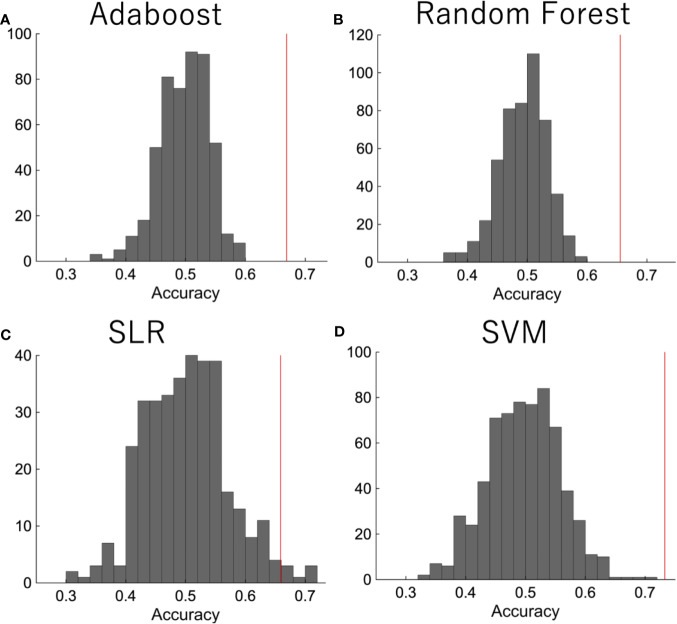
The permutation test. The diagnostic labels were randomly permuted for each subject and classification algorithms were applied, such as **(A)** random forest, **(B)** AdaBoost, **(C)** SLR, **(D)** SVM. The procedure was repeated 500 times.

We then did a permutation test in order to confirm that the performance was significant. The diagnostic labels were randomly permuted, and the performance of the classification was evaluated. The results showed that the classification accuracy was significantly higher than the change level for each algorithm (**Figure 3**).

In order to investigate the effects of site bias on the classification, the evaluation of classification was performed by leave-one-site-out cross-validation. The classification accuracies varied widely depending on the site (**Table 3**). The sensitivity and specificity especially were highly dependent on the site, which indicated that the classification was based on site bias rather than the feature associated with depression.

**Table 3:** Leave-One-Site-Out Cross-Validation.

site	site1	site2	site3	site4
accuracy	0.5	0.5	0.5	0.63
sensitivity	0	1	0	0.8
specificity	1	0	1	0.45

Subjects from three among four sites are used as the training data and their performance was evaluated using subjects from the remaining site. Area under the Receiver Operating Characteristic (ROC) Curve for Test Sites 1, 2, 3, and 4 are 0.71, 0.83, 0.66, and 0.73 respectively.

**Figure 4:**
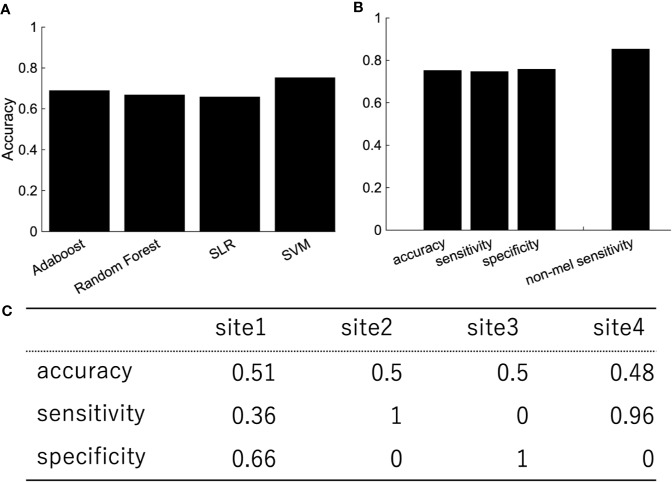
The performance of classification of melancholic patients and healthy controls **(A)** Accuracy, sensitivity, and specificity of the random forest by LOOCV. The rightmost bar is the accuracy rate that the melancholic classifier correctly classifies non-melancholic patients as depressed patients. **(B)** LOOCV performance of melancholic patients' classification using other classification algorithms. **(C)** Leave-one-site-out CV performance of melancholic patients' classification. Area under the ROC Curve for Test Sites 1, 2, 3, and 4 are 0.66, 0.5, 0.82, and 0.79, respectively.

Depression is heterogeneous. This could be the reason why the classification performance was affected by the site rather than the diagnostic label. We then focused on melancholic depression, which is a subtype of major depressive disorder with biological homogeneity (29–31). The classification performance between melancholic patients and healthy controls was slightly higher than the one between major depressed patients, including non-melancholic patients and healthy controls (**Figure 4**). The classifier classified the patients with non-melancholic depression with an accuracy of about 75.3%. The tendency of the accuracy depending on the algorithms was the same as in the case of the patients with major depressive disorder vs healthy controls. That is, SVM and ensemble learning showed good performance, whereas SLR did not. Moreover, the performance was evaluated by leave-one-site-out cross-validation. The classification accuracies varied widely depending on the site even without non-melancholic patients.

**Figure 5:**
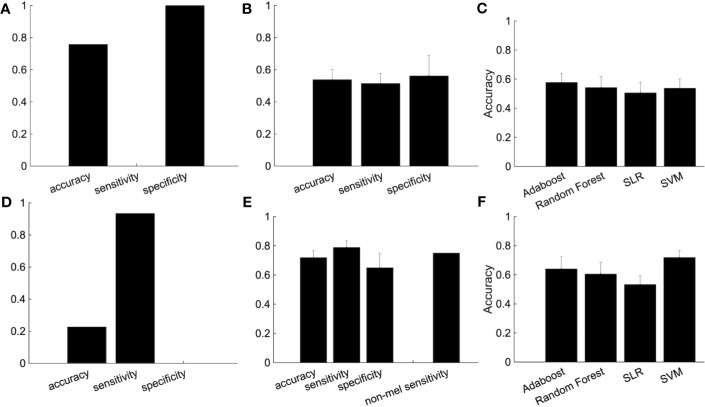
The improvement of the classification performance using the independent site. **(A)** All subjects from independent site data were used as test data. **(B)** The performance of the classification after part of the group of healthy subjects (not used as either the training or test data) are used for the regressing out. Because there are many combinations from which to choose healthy controls used for the regressing out, we randomly chose healthy controls for the regressing out, with procedure was repeated 100 times. The error bar indicated the standard deviation. **(C)** Performance of several classification algorithms. **(D-F)** The same as above but using only melancholic patients and healthy controls. **(E)** The rightmost bar is the accuracy rate that the melancholic classifier correctly classifies non-melancholic patients as depressed patients.

We then used subjects from the independent site (**Figure 5**). We trained the classifier using subjects from four sites and tested the classifier using subjects from the independent site. The performance shows the classification was based on site bias rather than the diagnostic label (**Figure 5A**). The decision boundary between patients and healthy controls obtained using training data is different from the boundary between patients and healthy controls of the test data. In order to overcome this problem, we used a part of the healthy controls from the independent site for regressing out the site, ages, and sex information, and the remaining healthy controls and depressed patients were used as test data. Note that the number of healthy controls and depressed patients used for the test data were set to be equal. Although the accuracy was 54.7%, this ingenuity reduced site bias (**Figure 5B**). Furthermore, when we focused on melancholic patients, the performance had improved drastically (**Figures 5D–F**) and the accuracy was 71.9%.

## Discussion

In this study, we proposed a method to reduce the site bias effect and improve the classification accuracy of patients with major depressive disorder for an independent test dataset. The most effective improvement was achieved by the calibration using a part of the independent dataset. Specifically, a part of the healthy controls group was used for regressing out site bias. Furthermore, when we focused on the classification between melancholic depressed patients and healthy controls, the effect of the calibration was further improved. In this case, SVM showed the best performance.

### Cooperation With Other Harmonization Methods

There are some studies trying to overcome site bias. Some studies used independent component analysis or sparse canonical component analysis to obtain site-independent brain activity (13). These methods are time-consuming, and it is possible that important features which characterize patients with depression are lost. On the other hand, regressing out is a simple way to reduce site bias and retains all features. While we employed a simple linear regression model for this purpose, it can be replaced by or combined with more sophisticated harmonization methods, such as combat, traveling subject methods, or generalized linear model (GLM) methods (1, 2, 14–18). Also, a non-linear model may be possible as done in (32), depending on the properties (i.e. the number of samples and sampling costs) of available data sets. Deciding when and for what should be selected among them is a future direction of this study.

### Heterogeneity of the Depressive Disorder

Depressive disorder is known to be heterogeneous. The melancholic depressive disorder is a typical and severe type of disorder. This would be a reason why the classification performance was improved when we focused on melancholic patients. Some critics suggest that overfitting may happen in this study because of subsampling a smaller number of subjects from the whole dataset. We sincerely accept the possibility. However, our study showed good performance even when we trained the model with the data set excluding non-melancholic patients and tested it with the independent data set including non-melancholic patients (**Figures 4B** and **5E**). The result indicates that it is important to apply a data cleansing process such that patients with atypical depression subtypes should be removed from the training data set. This process may serve to reduce uncertainty in the training data set to avoid the overfitting to depression-irrelevant factors. Targeting other types of depressive disorders would be future work. There are some studies about the subtyping the depressive disorder by using biological markers including functional connectivity (1, 6). If a new subtype of depressive disorder that showed characteristic neural behavior is found, the diagnosis for this subtype using machine learning would be improved.

### Factors Affecting Classification Performance

In the preprocess of the fMRI data, we conducted global signal regression (GSR). While the GSR can minimize global signal drifts, it may cause an additional bias by moving FC distribution to a negative direction (33–39). To investigate this possibility, we tried our proposed method excluding GSR and compared it with the method including GSR. As a result, the classification performance did not change so much (**Supplementary Figure 1**). Even though it was minor in our dataset, we have to bear in mind that GSR could be a factor to affect multi-site generalization.

We used Brainvisa Sulci Atlas to calculate the functional connectivity according to (7). Different parcellation will lead to different functional connectivity, resulting in a different prediction result. On the other hand, the main aim of our study is the generalization of developed diagnosis models rather than how to develop the best model suitable for a specific setting. To support this claim, we showed that our model was able to adjust the decision boundary between patients and healthy controls to improve the generalization (**Figure 5**).

Finally, we should bear in mind that the condition of medication may affect our classification performance. To minimize the medication effect, we designed this study so that all patients underwent the fMRI scan before or immediately (less than two weeks) after starting medication of selective serotonin reuptake inhibitors (SSRIs). However, acute neural effect of SSRIs was possible as reported in (40, 41), and we did not completely control the medication effect. To overcome this limitation, more sophisticated clinical trials will be required in our future study.

### Interaction Effects Between Sites and Features

In this study, we did not consider the interaction between site and other attributes such as age and sex. There is a possibility that the removal of the confounding bias could be more efficient if we consider the interaction. Actually, when we performed ANOVA for the interaction, the distribution of p-values of all FCs significantly differed from uniform distribution (Chi-square goodness-of-fit test, **Supplementary Figure 2**), implying that the interaction would be not neglectable. However, the inclusion of interaction increases the number of regression coefficients, which possibly leads to larger generalization errors, especially when the number of participants for calibration is limited. Assuming this scenario, we adopted a fixed effect regression in this study.

### For Clinical Diagnosis

To avoid overfitting, we employed a univariate feature selection method using a rank-sum test and filtered out non-significant FCs for the classification. While the method combined with a SVM classifier achieved the best generalization performance in our study, we should not conclude that all the selected FCs were the biomarkers crucial for depression diagnosis: The univariate feature selection method is apt to increase the risk of false positive more than the pre-designed significance level (i.e. the p-value threshold). We expected that SLR could be an alternative approach to coping with both generalization and feature selection simultaneously. However, the result was negative. Accordingly, the reasonable strategy to discover necessary and sufficient FCs for depression diagnosis is still an open question.

When we consider an application for clinical diagnosis, site bias is a large problem. If traveling subjects, who visit multiple sites to undergo fMRI with the same protocol, are available, we would be able to perform more effective calibration and thereby achieve better performance for the diagnosis of patients with MDD (2). Even if traveling subjects are not available, our proposed procedure would be helpful. Site bias should be calibrated using data from healthy subjects before clinical diagnosis, using the fMRI scanner used for the clinical diagnosis, which would be a more practical way for an application at a novel site.

## Data Availability Statement

The original data for this study will not be made publicly available due to the involvement of patient data. It can be obtained upon request to the Department of Psychiatry and Neurosciences, Hiroshima University, Japan (SY, yamawaki@hiroshima-u.ac.jp). IRB imposing these restrictions on our data is Ethical Committee for Epidemiology of Hiroshima University (contact: Shoji Karatsu kasumi-kenkyu@office.hiroshima-u.ac.jp). Additional contact information is available from: https://www.hiroshima-u.ac.jp/en/pharm/contact.

## Ethics Statement

The studies involving human participants were reviewed and approved by the Ethics Committee of Hiroshima University, the Radiation Drug Safety Committee, and by the institutional review board of the National Institutes for Quantum and Radiological Science and Technology. The patients/participants provided their written informed consent to participate in this study.

## Author Contributions

TN and JY carried out statistical analysis and gave interpretations. MT, NI, GO, YO, MY, TS, and SY designed a study, collected functional MRI data. TN and JY wrote the main manuscript text and prepared the figures with input from all authors.

## Funding

This research was supported by AMED under Grant Numbers JP19dm0107093 and JP19dm0107096, and Research on Medical ICT and Artificial Intelligence (H29-ICT-General-010), Health, Labour and Welfare Sciences Research Grants, Ministry of Health, Labour and Welfare Japan.

## Conflict of Interest

The authors declare that the research was conducted in the absence of any commercial or financial relationships that could be construed as a potential conflict of interest.
