# Supplementary material for: Enhancing Multi-Center Generalization of Machine Learning-Based Depression Diagnosis From Resting-State fMRI
Source: Front Psychiatry. 2020 May 28;11:400. doi: 10.3389/fpsyt.2020.00400 (PMC7270328; doi:10.3389/fpsyt.2020.00400)
Supplement: Supplementary file 1 [file Presentation_1.pptx]

## Slide 1
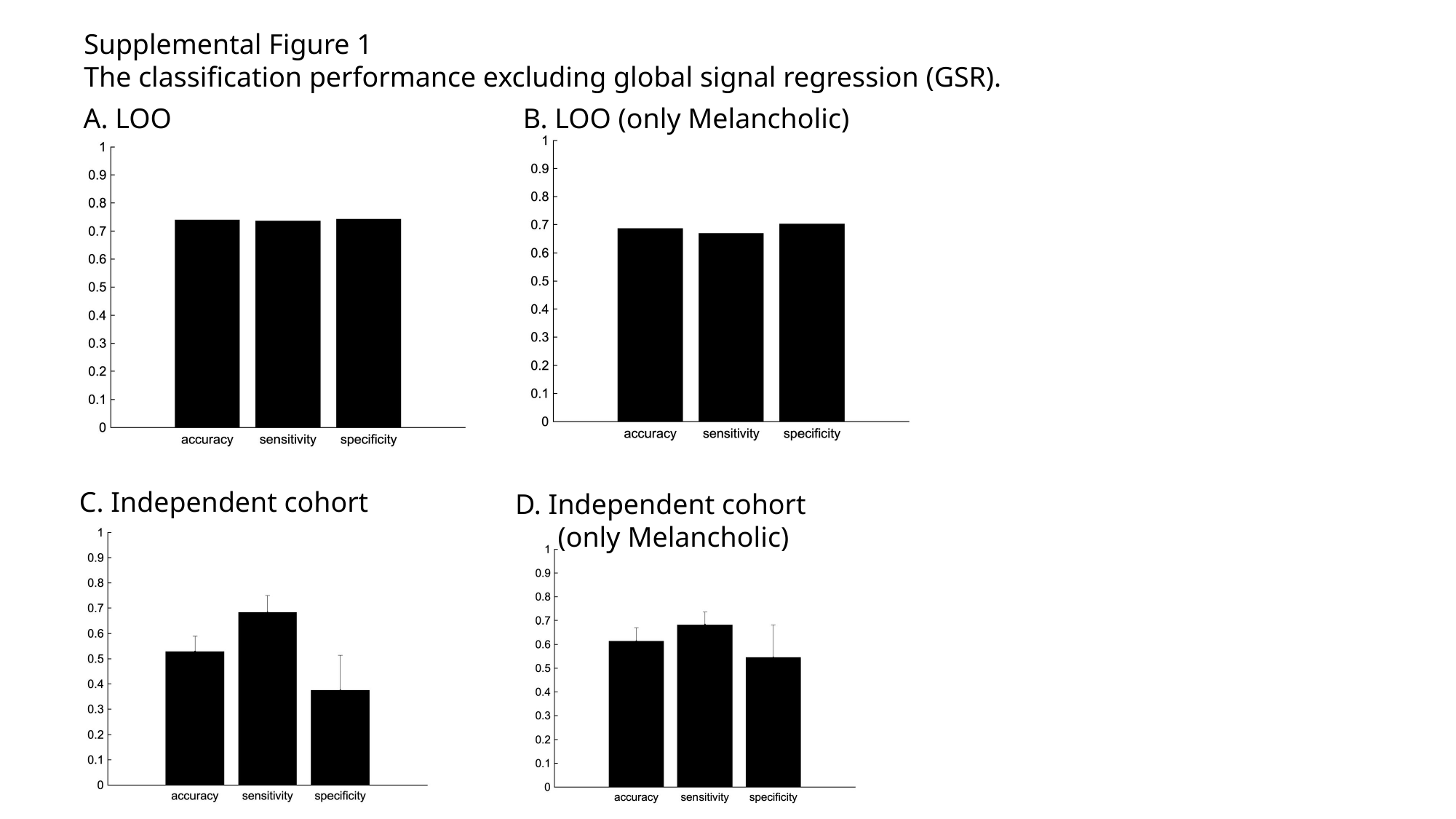

Supplemental Figure 1
The classification performance excluding global signal regression (GSR).
A. LOO
B. LOO (only Melancholic)
C. Independent cohort
D. Independent cohort
 (only Melancholic)

## Slide 2
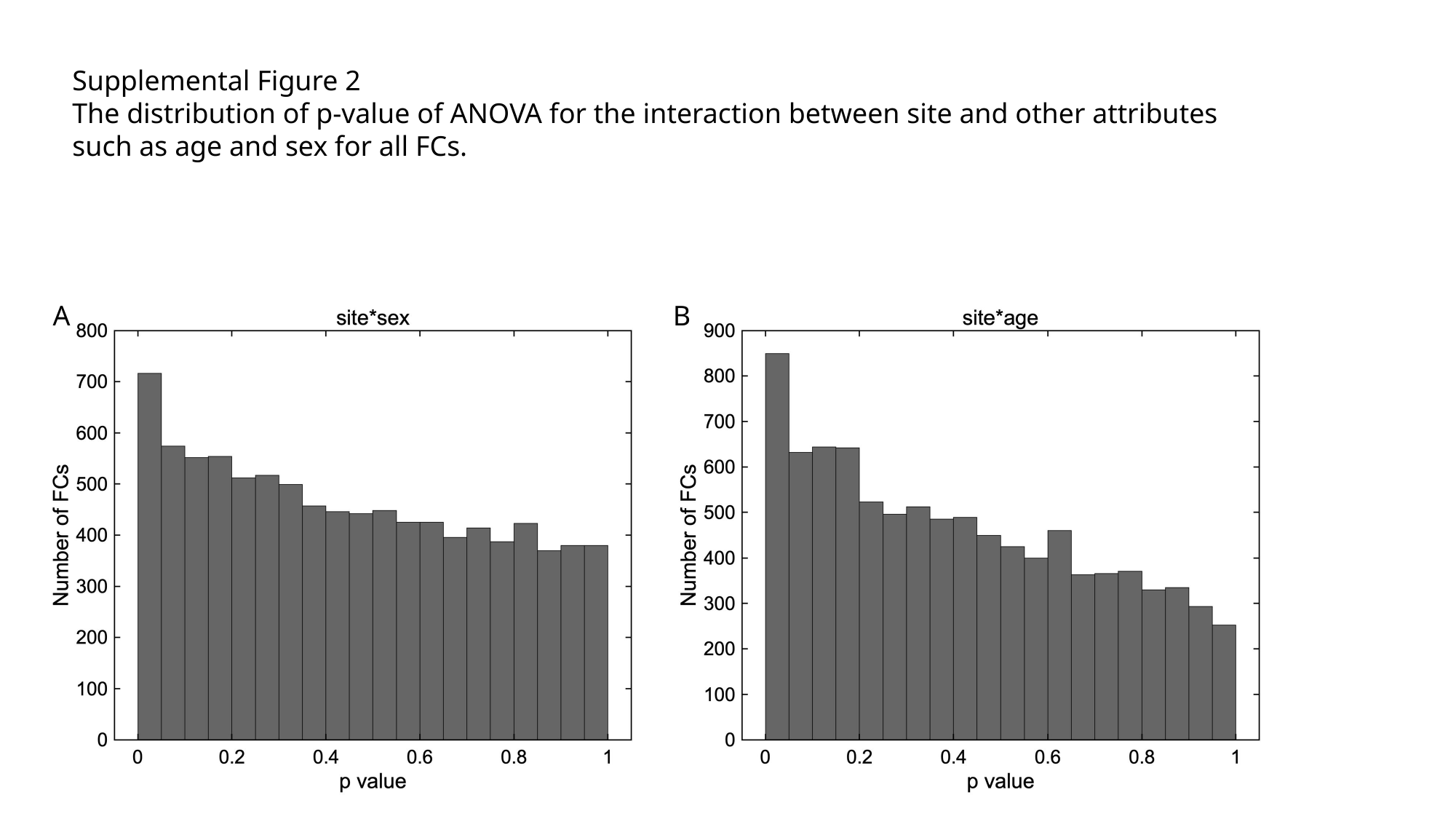

Supplemental Figure 2
The distribution of p-value of ANOVA for the interaction between site and other attributes such as age and sex for all FCs.
A
B
